# Histone H3.3 Variant Dynamics in the Germline of Caenorhabditis elegans

**DOI:** 10.1371/journal.pgen.0020097

**Published:** 2006-06-30

**Authors:** Siew Loon Ooi, James R Priess, Steven Henikoff

**Affiliations:** 1 Division of Basic Sciences, Fred Hutchinson Cancer Research Center, Seattle, Washington, United States of America; 2 Howard Hughes Medical Institute, Fred Hutchinson Cancer Research Center, Seattle, Washington, United States of America; Huntsman Cancer Institute, United States of America

## Abstract

Germline chromatin undergoes dramatic remodeling events involving histone variants during the life cycle of an organism. A universal histone variant, H3.3, is incorporated at sites of active transcription throughout the cell cycle. The presence of H3.3 in chromatin indicates histone turnover, which is the energy-dependent removal of preexisting histones and replacement with new histones. H3.3 is also incorporated during decondensation of the *Drosophila* sperm pronucleus, indicating a direct role in chromatin remodeling upon fertilization. Here we present a system to monitor histone turnover and chromatin remodeling during Caenorhabditis elegans development by following the developmental dynamics of H3.3. We generated worm strains expressing green fluorescent protein– or yellow fluorescent protein–fused histone H3.3 proteins, HIS-71 and HIS-72. We found that H3.3 is retained in mature sperm chromatin, raising the possibility that it transmits epigenetic information via the male germline. Upon fertilization, maternal H3.3 enters both male and female pronuclei and is incorporated into paternal chromatin, apparently before the onset of embryonic transcription, suggesting that H3.3 can be incorporated independent of transcription. In early embryos, H3.3 becomes specifically depleted from primordial germ cells. Strikingly, the X chromosome becomes deficient in H3.3 during gametogenesis, indicating a low level of histone turnover. These results raise the possibility that the asymmetry in histone turnover between the X chromosome and autosomes is established during gametogenesis. H3.3 patterns are similar to patterns of H3K4 methylation in the primordial germ cells and on the X chromosome during gametogenesis, suggesting that histone turnover and modification are coupled processes. Our demonstration of dynamic H3.3 incorporation in nondividing cells provides a mechanistic basis for chromatin changes during germ cell development.

## Synopsis

Germ cells carry genetic information from one generation to the next. They are converted to gametes during meiosis, which are then reprogrammed for development in the fertilized egg. Gamete production and developmental reprogramming involve dramatic changes in DNA packaging, but little is understood about how these changes are involved in resetting the developmental program for the whole organism. In spermatogenesis, DNA is stripped and repackaged into highly condensed chromatin. After fertilization, sperm DNA is again repackaged as it dramatically decondenses to fuse with the egg nucleus. These repackaging processes involve the four core histone proteins, which tightly wrap DNA into nucleosome particles. A universal variant form of histone 3, H3.3, is abundant in the germ cells of all plants and animals studied and has been shown to turn over at sites of active transcription in various somatic cells. The authors show that H3.3 displays dynamic turnover throughout germ cell development of the roundworm Caenorhabditis elegans. H3.3 incorporates during the first germline stem cell division, continues through meiosis, and ends up in sperm and eggs. Strikingly, H3.3 becomes depleted from primordial germ cells, and the meiotically silenced X chromosome is deficient in H3.3, which suggests that H3.3 dynamics during meiosis and reprogramming transmit epigenetic information.

## Introduction

Germ cell chromatin undergoes many dynamic remodeling events during gametogenesis and upon fertilization. In mammalian spermatogenesis, S phase histones are replaced by histone variants. The histone variants are then sequentially replaced by transition proteins and protamines, resulting in highly condensed and transcriptionally inert sperm DNA (reviewed in [1]). Sperm DNA is again remodeled upon fertilization, where it is rapidly decondensed, and protamines are replaced by histones to generate transcriptionally competent chromatin. During spermatogenesis, germ cell chromatin also undergoes other dynamic processes such as meiotic recombination, the formation of the XY body and establishment of genomic imprinting mediated by DNA methylation.

Histone variants, unlike S phase or replication-coupled (RC) histones, are expressed throughout the cell cycle and are incorporated into nucleosomes in a DNA replication–independent (RI) manner. In mammals, certain histone variants are expressed during different stages of gametogenesis; however, their specific functions during germ cell development have remained elusive (reviewed in [2,3]). H3.3 is a universal histone H3 variant that is incorporated at sites of active transcription [4–6]. H3.3 incorporation, which occurs in the form of H3.3/H4 dimers [7], likely accounts for the replacement of 20% to 30% of the RC histone H3. Thus, energy-dependent removal of preexisting histones and replacement by RI histones (histone turnover) is a widespread and dynamic process that can be measured by observing the presence of H3.3 in dividing cells. Dynamic incorporation of H3.3 might profoundly alter the chromatin state of the nucleosome by enriching it in post-translational modifications that are associated with active chromatin and depleting it in modifications associated with silent chromatin [8–10]. In addition, H3.3 is incorporated during decondensation of the *Drosophila* sperm pronucleus, indicating a direct role in chromatin remodeling upon fertilization [11].

To better understand histone turnover and chromatin remodeling during gametogenesis, we developed a system to analyze the spatial and temporal dynamics of histone H3.3 incorporation throughout development in Caenorhabditis elegans. C. elegans is transparent, making it an attractive organism for in vivo analysis of chromatin dynamics at any stage of development and in any cell type. Germ cells are particularly easy to visualize, because the gonad occupies almost half of the adult body, and successive stages of meiosis appear in a simple, linear sequence along the proximal-distal axis of the gonad. In this study, we generated worm strains expressing green fluorescent protein (GFP)- and yellow fluorescent protein (YFP)-tagged H3.3 and showed that the tagged H3.3 proteins are incorporated into nucleosomes. By observing fluorescently tagged H3.3 in living embryos, larvae and adult worms, we showed that H3.3 is associated with dynamic chromatin remodeling during multiple stages of germ cell development.

## Results

### The C. elegans Genome Encodes Two H3.3 and Three H3.3-Like Histone Variants

**Figure 1:**
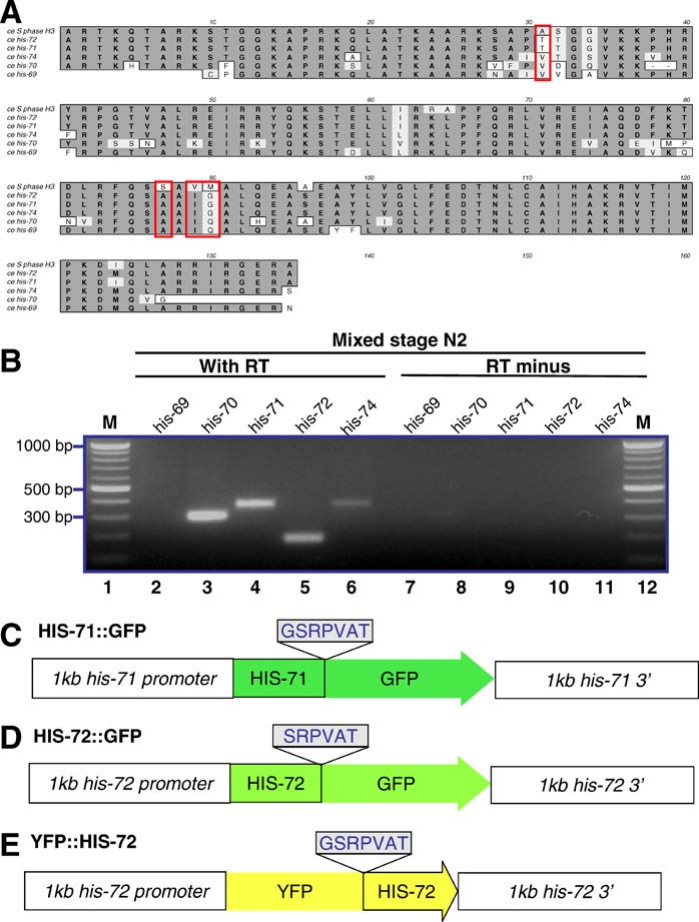
The C. elegans Genome Encodes Two H3.3 and Three H3.3-Like Histone Variants (A) Multiple sequence alignment of C. elegans H3, H3.3, and H3.3-like proteins. The red box denotes the four amino acids that are different between mammalian H3 and H3.3. (B) RT-PCR showed that *his-70, his-71, his-72,* and *his-74* are expressed in mixed stage N2 hermaphrodites. RT-PCR was performed with (lanes 2–6) or without (lanes 7–11) reverse transcriptase using gene-specific primers. Lanes 1 and 12 are DNA molecular standards. Diagrams of (C) *his-71::gfp,* (D) *his-72::gfp,* and (E) *yfp::his-72* transgenes used in this study. Linker amino acids shown were used to fuse GFP or YFP to HIS-71 and HIS-72. Each transgene included 1 kb upstream and 1 kb downstream of the coding sequence.

In animals, RC forms of histone H3 and the RI form (H3.3) differ at only four amino acids. At positions 31, 87, 89, and 90, RC H3 histones have amino acids ASVM, respectively, whereas H3.3 has SAIG. Residues 87, 89, and 90 of H3.3 are critical for RI nucleosome assembly [4], and phosphorylation of H3.3 S31 is specific to regions bordering centromeres in metaphase chromosomes [12]. To identify H3.3 genes within the C. elegans genome, we performed a tblastn search and found five high-scoring hits containing H3.3-specific residues (Figure 1A). These include four previously annotated histone genes: *his-69* (E03A3.3), *his-70* (E03A3.4), *his-71* (F45E1.6), and *his-72* (Y49E10.6). *his-69 *and *his-70* are predicted to encode N- and C-terminally truncated proteins, respectively. *his-70* may encode a protein lacking the C-terminal six to eight amino acids that was identified previously in total histone H3 purified from C. elegans [13]. *his-69* and *his-70* are tandem genes, and only one H3.3-like gene is present in the syntenic position in Caenorhabditis briggsae. The tblastn search revealed a fifth candidate H3.3 gene, which we call *his-74* (W05B10.1); the predicted HIS-74 is a highly diverged histone with no apparent C. briggsae counterpart.

To identify which H3.3 or H3.3-like genes are expressed, we analyzed RNA from mixed stage wild-type hermaphrodites using oligo dT primers and reverse transcription followed by PCR (RT-PCR). These experiments showed that *his-70, his-71, his-72,* and *his-74* are expressed (Figure 1B). However, we were not able to detect *his-69* mRNA from mixed stage wild-type hermaphrodites or an *him-8(e1489)* male–containing strain, even when *his-69*–specific primers were used for RT-PCR (unpublished data).

Based on sequence homology, HIS-71 and HIS-72 are the best candidates for functional H3.3 histone variants in C. elegans. Both have orthologs in C. briggsae and Caenorhabditis remanei. Both HIS-71 and HIS-72 have residues AIG at positions 87, 89, and 90 and a potentially phosphorylatable threonine at position 31. HIS-74 also contains residues AIG at positions 87, 89, and 90 but lacks a universally conserved phosphorylatable residue at position 31. HIS-71 and HIS-72 differ by only one amino acid, at position 124 (Ile and Met, respectively). *his-71* and *his-72* may be functionally redundant, because mutations in either *his-71 (tm1940)* or *his-72 (tm2066)* alone are homozygous viable (National BioResource Project, Tokyo, Japan).

### H3.3 Is Present in Chromatin throughout Development

To determine the spatial and temporal incorporation dynamics of H3.3 throughout development, we constructed transgenes encoding HIS-71 or HIS-72 fused at the C terminus to GFP (HIS-71::GFP and HIS-72::GFP) and HIS-72 fused at its N terminus to YFP (YFP::HIS-72) (Figure 1C–1E). Each transgene included 1 kb upstream and 1 kb downstream of the coding sequence.

**Figure 2:**
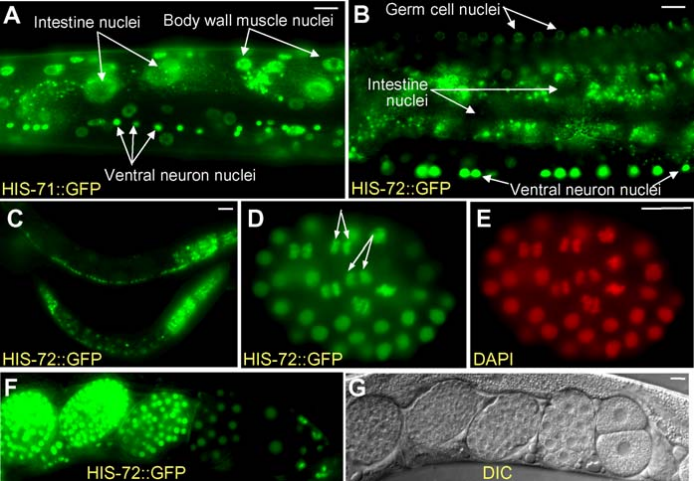
H3.3 Is Present throughout Development (A) HIS-71::GFP and (B) HIS-72::GFP fluorescence in living adults; cell types as indicated. Note absence of HIS-72::GFP in intestinal nuclei; small fluorescence particles surrounding the intestinal nuclei are autofluorescent gut granules. Note also that (B), the fluorescence of germ cell nuclei is less intense than that of somatic cell nuclei. (C) HIS-72::GFP expression in larvae; the bottom animal is an L1 larva. (D, E) Formaldeyde-fixed embryo showing HIS-72::GFP colocalization with DAPI staining. Double-headed arrows point to anaphase cells with characteristic bar-shaped structure of metaphase chromosome. (F, G) Fluorescence and DIC micrographs of embryos at various stages expressing HIS-72::GFP; the embryo at the right is at the two-cell stage. Scale bars, 10 μm.

Worm strains containing each transgene were generated, and living and fixed embryos, larvae, and adults were examined by fluorescence microscopy (Figure 2A–2G). HIS-71::GFP displayed high levels of expression in almost all adult nuclei (Figure 2A). HIS-72::GFP displayed a similar expression pattern in almost all larval and adult nuclei (Figure 2B and 2C), except that intestinal nuclei showed only low levels of expression (Figure 2B). Another apparent difference between HIS-71 and HIS-72 was seen during the onset of embryonic expression. With the *his-71::GFP* transgene, fluorescence was detected at the approximately 51-cell stage (embryos with four E blastomeres) (unpublished data), whereas in crosses where sperm bearing the *YFP::his-72* transgene fertilized wild-type oocytes, fluorescence from embryonic expression was detected at the approximately 26-cell stage (embryos with two E blastomeres) (see below).

**Figure 3:**
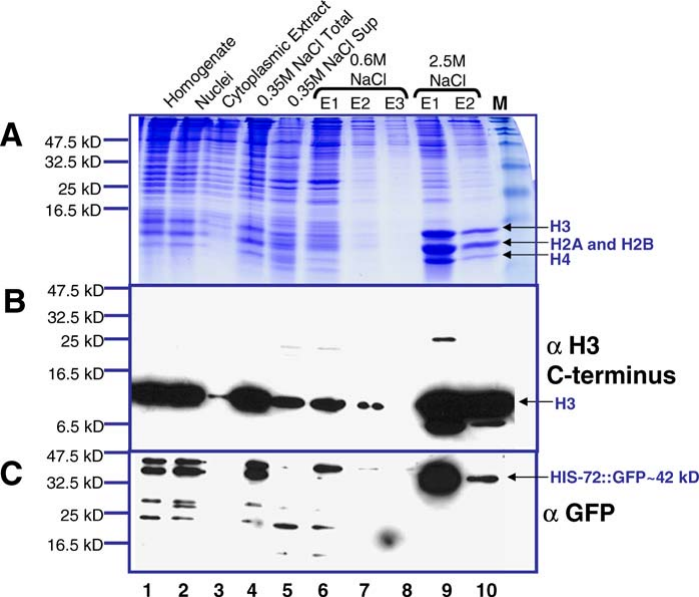
HIS-72::GFP Is Incorporated into Nucleosomes Histones were extracted with increasing salt concentrations from embryos expressing HIS-72::GFP. Core histones elute at high (2.5 M NaCl) but not low salt concentration. HIS-72::GFP elutes primarily at 2.5 M NaCl. (A) Coomassie blue staining and Western blot analysis using (B) a histone H3 antibody (ab1791 from Abcam) and (C) a GFP antibody (ab6556 from Abcam) for different steps during core histone extractions. Note that the histone H3 antibody (ab1791) does not recognize HIS-72::GFP. This could be because the epitopes recognized by ab1791 is IQLARRIRGERA, whereas the sequence of HIS-72 is MQLARRIRGERA. Nuclei and a low amount of insoluble proteins were carried over to the salt extraction lanes. Nevertheless, high salt extraction was still able to separate core histones from chromatin-associated proteins. Lanes 1, homogenized embryos; 2, nuclei; 3, cytoplasmic extract; 4, nuclei resuspended in 0.35 M NaCl; 5, supernatant of centrifuged nuclei resuspended in 0.35 M NaCl; lanes 6, 7, and 8: the first, second, and third elutes of nuclei pellet resuspended in 0.6 M NaCl, respectively; lanes 9 and 10, the first and second elutes of nuclei resuspended in 2.5 M NaCl, respectively; and M, protein molecular standard.

In embryos, HIS-72::GFP can be detected in nuclei at all stages (Figure 2F and 2G). When fixed nuclei were viewed at high magnification, the most intense HIS-72::GFP and YFP::HIS-72 fluorescence appeared coincident with DAPI-stained DNA at all stages of the cell cycle (Figure 2D and 2E). For example, mitotic chromosomes coalesce into a characteristic bar-shaped structure at metaphase that displayed high levels of fluorescence (Figure 2D and 2E). These observations suggest that the tagged H3.3 proteins are incorporated into nucleosomes. To test this possibility in the *his-72::gfp* strain, we used a high salt histone extraction method to separate nucleosomes (containing histone octamers) from nonnucleosomal proteins [14]. Extracts from lysed embryonic nuclei were bound to hydroxyapatite and eluted at increasing salt concentrations. Nonnucleosomal proteins are predicted to elute in 0.35 M NaCl, and core histones elute in 2.5 M NaCl. Coomassie blue staining and Western blot analysis using an anti-histone H3 antibody confirmed that C. elegans histones are enriched in the 2.5 M NaCl eluate fractions (Figure 3A and 3B). The majority of HIS-72::GFP, predicted to be about 42 kDa, stained positively with an anti-GFP antibody in the 2.5 M NaCl eluate fractions, indicating that HIS-72::GFP is incorporated into nucleosomes (Figure 3C). Our combined results suggest that H3.3 is present in chromatin throughout development.

### H3.3 Is a Component of Mature Sperm Chromatin

**Figure 4:**
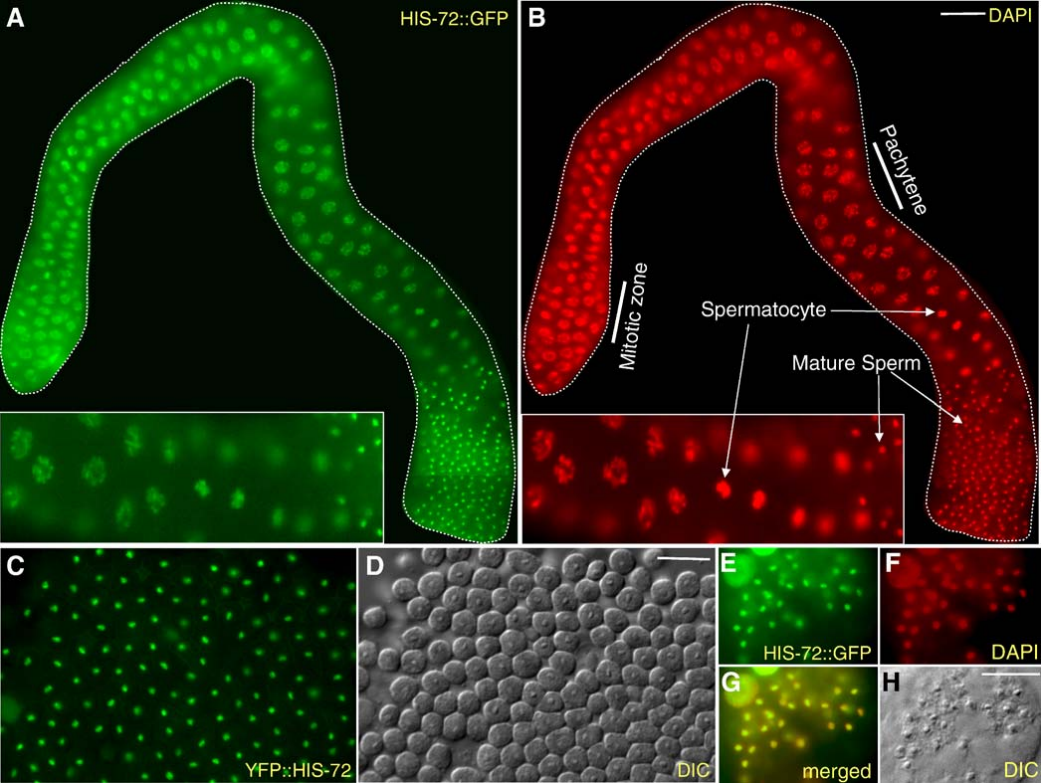
H3.3 Is a Component of Mature Sperm Chromatin (A, B) HIS-72::GFP is present throughout the male gonad starting from the mitotic zone and colocalizes with DAPI-staining; insets show enlarged regions of the gonads. (C, D) YFP fluorescence and DIC images of sperm from a dissected male. (E–H) Sperm from a dissected and formaldehyde-fixed hermaphrodite showing HIS-72::GFP and DAPI colocalization. Scale bars, 10 μm.

Previous immunostaining experiments in Drosophila melanogaster failed to detect H3.3 in developing sperm beyond the spermatocyte stage, suggesting that H3.3 is not present in mature sperm [15]. However, high-performance liquid chromatography has provided evidence of H3.3 in human sperm [16]. We readily detected HIS-72::GFP and YFP::HIS-72 by fluoresecence at all stages of C. elegans spermatogenesis (Figure 4A and 4B). HIS-72::GFP colocalizes with DAPI in pachytene stage nuclei and in the single chromatin mass characteristic of spermatocytes in both males and hermaphrodites. Surprisingly, both YFP::HIS-72 and HIS-72::GFP are retained in mature sperm in both male and hermaphrodite worms (Figure 4C–4H). HIS-72::GFP colocalizes with DAPI, showing that HIS-72::GFP is incorporated into sperm chromatin (Figure 4E–4H). We observed similar incorporation patterns during spermatogenesis in hermaphrodite worms. We were unable to detect HIS-72::GFP by immunostaining in C. elegans spermatocytes or mature sperm. The highly compact sperm chromatin may limit antibody accessibility, making it difficult to detect H3.3 or HIS-72::GFP by immunostaining. Because H3.3 is present in the sperm of both nematodes and humans, we propose that H3.3 retention is likely to be a general feature of animal spermatogenesis.

### H3.3 Is Detected throughout Oogenesis

**Figure 5:**
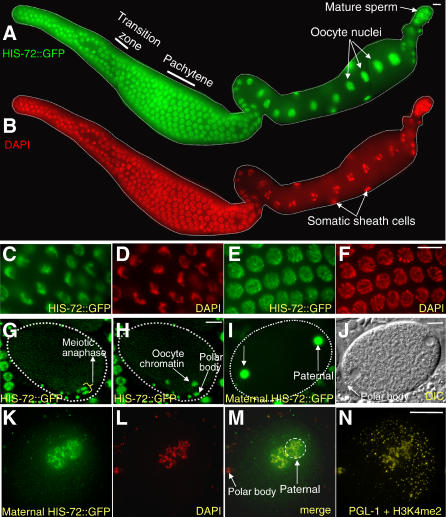
H3.3 Is Provided Maternally in Oocytes and Maternal H3.3 Is Incorporated into Paternal Chromatin upon Fertilization (A, B) HIS-72::GFP colocalizes with DAPI staining throughout the oogenic gonad. High magnification images of the (C, D) transition zone and (E, F) pachytene region. In the oocyte, HIS-72::GFP is provided maternally in the nuclei. (A, C, E) GFP fluorescence and (B, D, F) DAPI images of gonads dissected from hermaphrodites. (A) and (B) are composite images. (G, H) Times-lapse images of a single oocyte at meiosis II as the maternal chromosomes divide to segregate a polar body. (I–N) N2 males were crossed to temperature-sensitive *fem-1(hc17*ts*) unc-4(e120)* HIS-72::GFP hermaphrodites grown at nonpermissive temperature (23 °C). Upon fertilization, as soon as the nuclear envelope of the two pronuclei are formed, (I, J) maternal HIS-72::GFP is imported into both pronuclei and (K–N) incorporated into paternal chromatin. (I, J) GFP fluorescence and DIC images of a live, in utero one-cell embryo. (K–N) Images of a single, dissected, and methonal-fixed one-cell embryo labeled as shown. The maternal pronucleus can be identified by its proximity to the polar body (J and M), and the male pronucleus by its proximity to posterior-localized PGL-1 protein (N). In (N), PGL-1 localization is cytoplasmic in the zygote, while H3K4me2 is localized to chromatin. Scale bars, 10 μm.

Next, we analyzed the incorporation of HIS-72::GFP in oogenic germ cells. HIS-72::GFP was detected in germ cell nuclei throughout all stages of oogenesis (Figure 5A–5F). HIS-72::GFP expression was largely coincident with that of DAPI-stained chromatin. For example, the DNA in germ nuclei exiting mitosis and entering meiosis (transition zone nuclei) has a characteristic crescent shape that appears identical by both DAPI staining and HIS-72::GFP fluorescence (Figure 5C and 5D). During the diakinesis stage of meiosis, oocytes greatly enlarge in size and in nuclear volume. In these enlarged nuclei, HIS-72::GFP is present at high levels throughout the nucleoplasm (Figure 5A and 5B), which would obscure any chromatin-localized HIS-72::GFP. Indeed, less stringent fixation conditions that allowed some diffusion of the nucleoplasm revealed chromosome-associated HIS-72::GFP in mature oocytes (see below). In addition, we observed that HIS-72::GFP is chromatin-localized in fertilized oocytes completing meiosis I and II (Figure 5G and 5H). We hypothesize that oocytes synthesize high levels of H3.3 in preparation for embryogenesis.

### Maternal H3.3 Is Incorporated into Both Pronuclei and the Paternal Chromatin upon Fertilization

We wanted to determine whether maternally provided H3.3 might remodel the paternal chromatin once oocytes are fertilized. At fertilization, the sperm chromatin decondenses and acquires a nuclear envelope (forming the paternal pronucleus). A separate nuclear envelope surrounds the maternal chromosomes (the maternal pronucleus), and the two pronuclei migrate toward the center of the egg where they fuse into a single large nucleus. These initial events of embryogenesis are believed to occur in the absence of embryonic transcription [17,18]. To assay the fate of maternally provided H3.3, we introduced the *his-72::GFP* transgene into temperature-sensitive *fem-1(hc17ts) unc-4(e120)* hermaphrodites that produce oocytes, but not sperm, when cultured at a nonpermissive temperature (23 °C). Mating these worms with wild-type males yields one-cell embryos that contain only maternally synthesized HIS-72::GFP. We found that maternal HIS:72::GFP enters both the paternal and maternal pronuclei as soon as they are formed (Figure 5I and 5J). HIS-72::GFP showed both a nucleoplasmic and chromosomal localization pattern, suggesting that it had incorporated into the paternal chromatin (Figure 5K–5N). Because HIS-72::GFP was already present on oocyte chromatin during oocyte meiotic maturation, we could not determine if additional maternal HIS-72::GFP is incorporated into maternal chromatin upon fertilization. We conclude that maternally provided HIS-72 may remodel paternal chromatin before the first cell division and prior to the onset of transcription.

H3.3 within sperm chromatin might be retained in the paternal chromosomes after fertilization, thus providing an inherited epigenetic marker. To address this issue, we crossed males expressing HIS-72::GFP to feminized *fem-1(hc17*ts*) unc-4(e120)* hermaphrodites lacking the transgene. After mating, large numbers of GFP-containing sperm were visible in the uterus and spermatheca of the hermaphrodite (Figure S1). The GFP fluorescence associated with individual sperm disappeared very soon after fertilization, and no fluorescence was visible on the paternal chromosomes as the paternal pronucleus formed. Taken together with the abundance of chromatin-bound maternal HIS-72::GFP in the paternal pronucleus, this disappearance demonstrates that most of the H3.3 in paternal chromatin is maternally derived. Whether or not a low amount of paternally derived H3.3 is retained postfertilization could not be determined, because decondensation of sperm resulted in a marked dilution of the signal.

### The X Chromosome Is Deficient in H3.3 during Gametogenesis

The adult male (XO) and hermaphrodite (XX) gonads contain rows of germ nuclei in sequential stages of mitosis and meiosis. Previous studies have shown that X chromosomes differ from autosomes in histone modifications during gametogenesis in C. elegans and mice [19–21]. Histone H3 K9 dimethyl (H3K9me2), a histone modification associated with transcriptional repression, is enriched on the meiotically silenced XY body of male mice [20]. In C. elegans males, H3K9me2 is similarly enriched on the X chromosome at the pachytene stage, suggesting that this chromosome is also meiotically silenced [19,21]. In the mitotic zone and in the pachytene stage of meiosis, the single X chromosome in males and the two X chromosomes in hermaphrodites are deficient in histone modifications associated with transcriptional activity, such as histone H3 K4 dimethyl (H3K4me2) [19,21]. Only in postpachytene stages of oogenesis do the X chromosomes begin to accumulate histone modifications associated with transcriptional activity [19,21], coincident with a burst of expression of X-linked genes involved in oogenesis [22].

**Figure 6:**
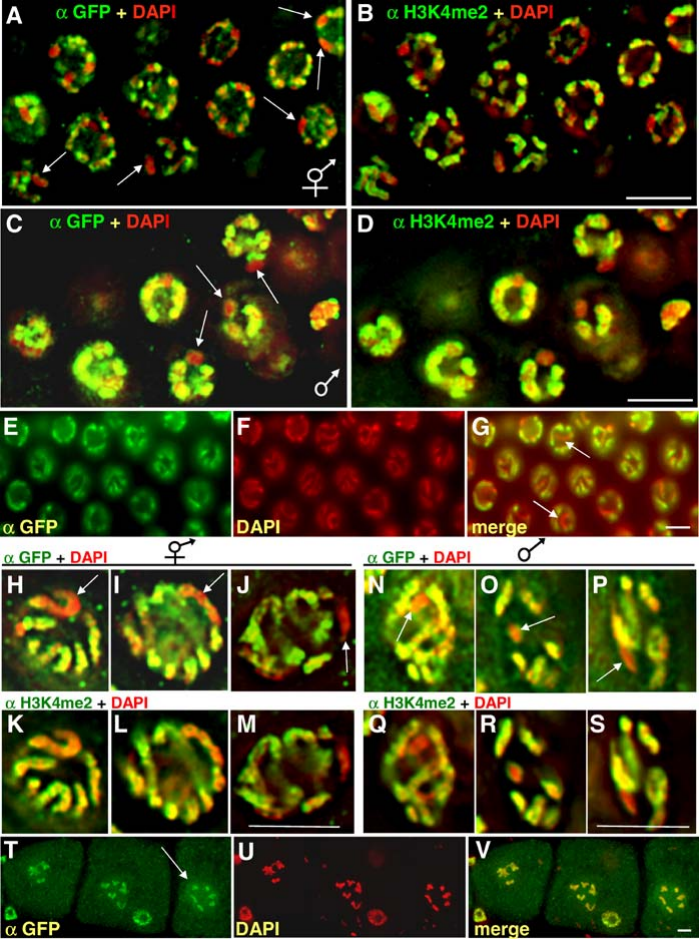
The X Chromosome Is Deficient in H3.3 during Gametogenesis Mitotic regions of adult (A, B) hermaphrodite oogenic and (C, D) male gonads fixed and stained for GFP, H3K4me2, and DNA. Arrows indicate chromosomes lacking both YFP::HIS-72 and H3K4me2. (E–G) Pachytene region of adult hermaphrodite fixed and stained for GFP and DNA. Note that one chromosome pair lacks both YFP::HIS-72 and H3K4me2. (H–S) High magnification images of single pachytene stage nuclei from a (H–M) wild-type hermaphrodite or a (N–S) heterozygous *his-72(tm2066)* male fixed and stained as indicated. (T–V) Oocytes at diakinesis showing YFP::HIS-72 localization to each of the six paired chromosomes (arrow points to a single nucleus). Scale bars, 5 μm.

In our analysis of H3.3 in the hermaphrodite and male germlines, we found that the mitotic zone and pachytene stage nuclei showed nonuniform localization of YFP::HIS-72 (Figure 6A–6S). In general, chromosomes showed varying levels of YFP::HIS-72 along their length. However, two entire chromosomes were consistently deficient in YFP::HIS-72 in the mitotic nuclei of hermaphrodites (Figure 6A), and one chromosome was deficient in YFP::HIS-72 in the mitotic nuclei of males (Figure 6C). The same chromosomes were also deficient in H3K4me2 (Figure 6B and 6D, respectively). During the pachytene stage of meiosis, one set of paired chromosomes was deficient in YFP::HIS-72 (Figure 6E–6G) in hermaphrodites. In costaining experiments, we found that the same chromosomes that were deficient in YFP::HIS-72 were also deficient in H3K4me2, confirming that they are the X chromosomes (Figure 6H–6M and Video S1). Similarly, in the pachytene stage of male spermatogenesis, one chromosome was deficient in YFP::HIS-72, and this chromosome also showed reduced staining for H3K4me2 (Figure 6N–6S and Video S2). By the diakinesis stage of oogenesis, HIS-72::GFP can be detected uniformly on all chromosomes under mild fixation conditions in which nucleoplasm is allowed to diffuse away (Figure 6T–6V). We conclude that the X chromosome, when compared to the autosomes, undergoes very little histone turnover in germ cells. Because studies in other systems have shown that H3.3 is enriched in “active” modifications such as H3K4me2 [8–10], our results suggest that histone H3.3 turnover and H3K4me2 modification are coupled processes.

### H3.3 Becomes Depleted from Primordial Germ Cells

The C. elegans germline originates from a series of asymmetric, stem cell–like divisions in the early embryo; this lineage of cells is called the P lineage (reviewed in [23]). After a P lineage cell divides, one daughter produces only somatic cell types and the other daughter becomes the new P lineage cell, named in succession P_1_, P_2_, P_3_, and P_4_. After the final asymmetric division, P_4_ divides equally into two primordial germ cells called Z2 and Z3; these cells do not divide again during embryonic development, but proliferate in larvae to produce all of the germ cells.

**Figure 7:**
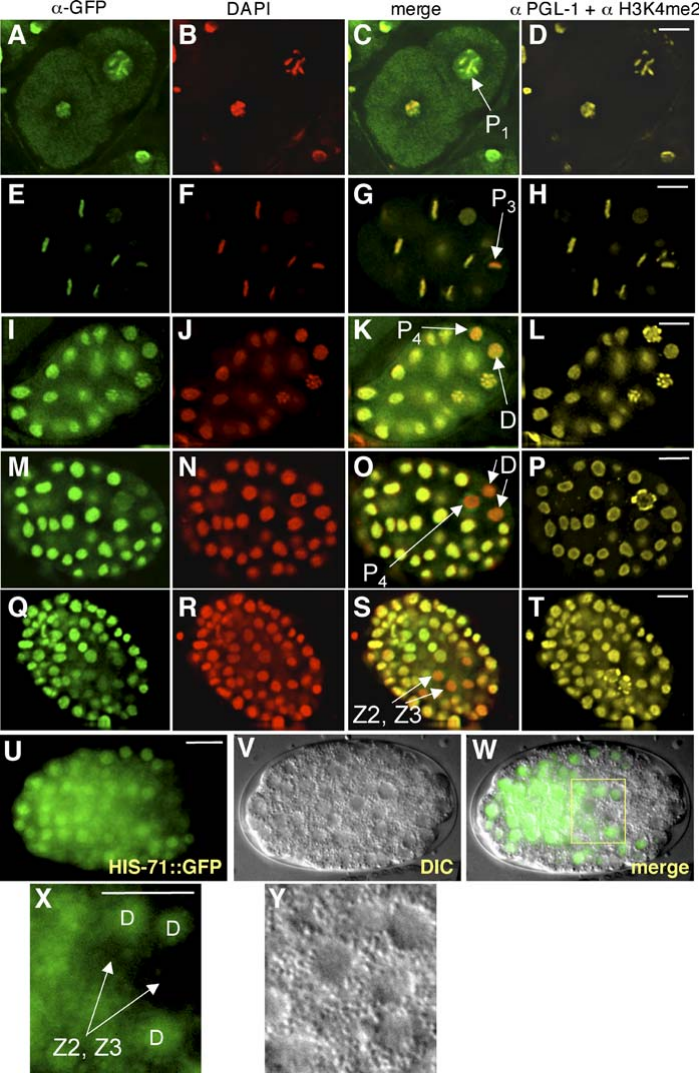
H3.3 Becomes Depleted from the Primordial Germ Cells (A–T) Each row shows successively older HIS-72::GFP-expressing embryos fixed and stained as indicated in the column headings; the top row is a two-cell embryo and row (M–P) is a 80- to 90-cell stage embryo. P lineage cells are indicated in the merge column. Note the relative loss of HIS-72::GFP in the older P lineage cells and in (Q–T) Z2 and Z3. The nearest relatives of P_4_ (the D cell and its descendants, labeled) also show a relative lack of HIS-72::GFP. (U–Y) HIS-71::GFP expression in a live embryo; (X) and (Y) are high magnifications of the boxed area in (W), showing the Z2, Z3, and D cells. Note that HIS-71::GFP is expressed at relatively low levels in Z2 and Z3 but is expressed in D descendants at levels comparable to other somatic precursors. Scale bars, 10 μm.

We found that HIS-72::GFP can be detected in each of the P_1_, P_2_, P_3_, and P_4_ cells and their somatic precursor sister cells (Figure 7A–7T). However, the level gradually decreases in the P lineage. For example, metaphase P_3_ chromatin displays less HIS-72::GFP staining when compared to chromatin of somatic blastomeres (Figure 7E–7H). By the 80- to 90-cell stage, very little HIS-72::GFP was visible in P_4_. While HIS-72::GFP was not detectable in the somatic sister of P_4_, called the D cell, or the D daughters (Figure 7M–7P), HIS-71::GFP was present in later D descendants (Figure 7U–7Y). Levels of both HIS-71::GFP and HIS-72::GFP remained low in Z2 and Z3 during later embryonic stages (Figure 7Q–7Y), although the larval descendants of Z2 and Z3 that proliferate to form the germ cells have detectable levels of H3.3 (unpublished data).

**Figure 8:**
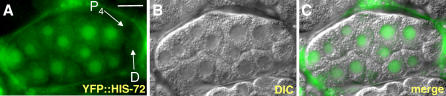
Embryonic YFP::HIS-72 Expression Is Absent from P_4_ and D Cells (A–C) YFP::HIS-72 expression in a live, approximately 26-cell stage embryo. The male parent of the embryo carried the *yfp::his-72* transgene, but the maternal parent did not. The transgene is not expressed in the P lineage cell P_4_ or in the D cell. Scale bars, 10 μm.

Cells in the embryonic P lineage appear to lack RNA polymerase II–dependent transcription [17]; however, transcription is initiated in the somatic sisters of these cells after division. Therefore, a simple explanation for the presence of HIS-72::GFP in the early P lineage cells, and the absence in later P lineage cells, would be the depletion of maternal HIS-72::GFP combined with the absence of embryonic *his-72::gfp* transcription. To address when embryonic transcription of H3.3 begins in the P lineage, we crossed males expressing either the *his-72::gfp* or *yfp::his-72* transgene into wild-type worms. While somatic cells expressed both transgenes at high levels, no expression was seen in the P lineage or in the D cell (Figure 8A–8C and unpublished data). Thus, maternally synthesized, but not embryonically expressed, H3.3 is incorporated into the early germline blastomeres; this maternal H3.3 gradually disappears from the P lineage and becomes specifically depleted from primordial germ cells during embryogenesis.

## Discussion

We have introduced a system to visualize histone variant H3.3 dynamics in living C. elegans. We have identified HIS-71 and HIS-72 as histone H3.3 variants and have shown that HIS-72::GFP incorporates into nucleosomes. Strains harboring the *his-72* transgene are particularly useful for visualizing chromatin dynamics in the germline. A transgene encoding H1.1::GFP was described previously in *C. elegans;* however, this transgene showed germline expression in only about 2% of animals [24]. In contrast, HIS-72::GFP and YFP::HIS-72 are expressed at high levels throughout gametogenesis in both males and hermaphrodites (approximately 90% and approximately 70% of animals, respectively).

Using this system, we observed H3.3 incorporation throughout embryogenesis, during larval stages and in adult worms. In early embryos, H3.3 is present in gradually decreasing amounts in the germline P lineage (P_0_ to P_4_). The P lineage is unusual in that it undergoes stem cell–like divisions, and P_0_ to P_4_ cells are largely transcriptionally quiescent. After the final P lineage cell (P_4_) divides to yield Z2 and Z3 primordial germ cells, H3.3 remains deficient, and this deficiency persists during embryogenesis. We could not detect embryonic incorporation of H3.3 in the P lineage when the transgene was provided only through male sperm, consistent with previous findings that the P lineage appears to be transcriptionally quiescent, based on RNA polymerase II and transcript analysis [17,18]. Therefore, H3.3 in the early P lineage cells must be maternally derived. Because early P lineage cells contain H3.3, the observation that Z2 and Z3 lack H3.3 could mean that chromatin-associated H3.3 is selectively removed, or is simply diluted out by successive cell divisions. The deficiency of H3.3 in Z2 and Z3 cannot be attributed to a lack of transcription entirely, because some transcription evidently occurs in these cells by mid-embryogenesis, and an RNA polymerase II C-terminal domain phosphoserine 2 modification that correlates with transcriptional elongation is present in Z2 and Z3 [17,25,26].

During larval stages and in adult worms, H3.3 is present throughout both oogenesis and spermatogenesis, starting from the mitotic zone before meiosis begins, eventually ending up in mature oocytes and sperm. However, our analysis of germ cells in the adult gonad shows that the levels of H3.3 are not equivalent on all chromosomes. Instead, the X chromosomes are deficient in H3.3 compared to the autosomes, suggesting that the X chromosomes have a low histone turnover rate. Taken together with our observation that the two primordial germ cells of early embryos are deficient in H3.3, the asymmetry in histone turnover between the X chromosome and autosomes is most likely established during germ cell proliferation in larval stages.

Our observations on H3.3 expression in the primordial germ cells and in germ cells undergoing gametogenesis are in striking parallel with previous observations of histone modifications associated with transcriptional activity [19,21]. First, both H3K4me2 and H3.3 are present at very low levels in Z2 and Z3 during embryogenesis but at high levels in the Z2 and Z3 descendants as these cells proliferate during larval development. Second, in the mitotic region of the adult gonad the X chromosomes of males and hermaphrodites are deficient in both H3K4me2 and H3.3. Third, at the pachytene stage of meiosis the paired X chromosomes of hermaphrodites and the unpaired X of males are also deficient in both H3K4me2 and H3.3. Finally, similar high levels of both H3K4me2 and H3.3 are present on X chromosomes and autosomes during postpachytene stages of oogenesis. Low histone turnover may result from a lack of transcription-coupled chromatin remodeling on the X chromosomes, which are depleted of germline- and spermatogenesis-specific genes [22]. Considering that H3.3 is enriched in “active” modifications [8–10], our results suggest that deficiencies in H3K4 methylation and other H3 and H4 modifications result from reduced histone turnover, which involves replacement of H3/H4 with H3.3/H4.

The single X chromosome of male worms resembles the XY body of male mice in that they both have highly condensed structures and are meiotically silenced. Inactivation of the spermatocyte's X chromosome may also have consequences beyond meiosis, because in mice the paternal X chromosome is specifically inactivated in extraembryonic tissues [27]. Although we were unable to score individual chromosome during the final stages of spermatogenesis, we speculate that the X chromosome in mature sperm remains deficient in H3.3. Our observation that the C. elegans male X chromosome is deficient in H3.3 raises the possibility that reduced histone turnover is involved in maintenance of this chromosome-specific epigenetic state. Epigenetic events have been documented in C. elegans [28–30]. For example, some transgenes are imprinted for expression in the male germline [30], and perhaps expression level is correlated with the amount of H3.3 that accumulates on the transgene locus during spermatogenesis.

Another possible impact of H3.3 incorporation on the maintenance of an epigenetic state comes from our observation that it is retained in mature sperm of both males and hermaphrodites. This retention is reminiscent of the observation that the centromere-specific histone 3 variant, CENP-A, is quantitatively retained in bull sperm [31]. CENP-A–containing nucleosomes are thought to provide a foundation for maintaining centromeres epigenetically through mitosis and meiosis, so that CENP-A retention in bull sperm implies a role in epigenetic transmission. Likewise, our discovery that the other universal RI histone 3 variant, H3.3, is also retained in mature sperm, suggests an analogous role in epigenetic transmission that might in the future be explored using the powerful tools available for C. elegans.

Upon fertilization, maternal H3.3 incorporates rapidly into both maternal and paternal pronuclei of C. elegans. This is consistent with the previous observation that H3K4me2 is detected in both pronuclei [28], and suggests that H3.3 incorporation may be responsible for the appearance of H3K4me2. Our observation that H3.3 is incorporated into paternal chromatin is consistent with the detection of both H3.3 and its chaperone, HIRA, in the male pronucleus of Drosophila melanogaster [11]. Similarly, mouse HIRA is preferentially incorporated into the paternal pronucleus upon fertilization [32]. The difference between worms, in which both pronuclei incorporate H3.3, and flies and mice in which only the paternal genome is remodeled, might stem from a need to distinguish maternal from paternal genomes in flies and mice, but not in worms. In support of this interpretation, we note that mouse maternal and paternal genomes differ in global DNA methylation and histone modifications in the zygote, which are thought to result in parental imprinting (reviewed in [33]).

The C. elegans genome appears to be transcriptionally quiescent in one-cell embryos, because the elongation form of RNA polymerase II and newly transcribed mRNAs are first detected in somatic blastomeres at the four-cell stage [17,18,34]. Thus, our observation that H3.3 is incorporated into paternal chromatin upon fertilization in one-cell embryos provides additional evidence that H3.3 can be deposited independent of transcription [11]. Because H3.3 is the predominant form of histone 3 available throughout cell cycle, it most likely serves as a chromatin repair protein that is incorporated during chromatin-related events outside of S phase, such as chromatin remodeling upon fertilization and DNA repair.

In conclusion, our description of histone H3.3 dynamics during multiple stages of germ cell development implicates chromatin remodeling in germline maintenance and transmission. C. elegans entirely lacks DNA methylation and so provides an ideal model for studying purely histone-based epigenetic processes. This system, in conjunction with the powerful genetics of C. elegans, provides a unique opportunity to dissect chromatin dynamics during fertilization and other rapidly unfolding events in real time.

## Materials and Methods

### Nematode strains and maintenance.

Nematodes were cultured and manipulated genetically as described [35]. All strains were grown at 20 to 23 °C. The wild-type strain was the N2 (Bristol) strain. The following mutant alleles and strains were used: LGII: *unc-4(e120)*; LGIII: *unc-119(ed3); his-72* (*tm2066,* a gift from National BioResource Project, Tokyo, Japan); and LGIV: *fem-1(hc17ts)*. The following transgenic strains were created for this study: zuEx182 [(*his-71* ^1kb^::HIS-71::GFP); *unc-119(ed3)*], zuIs178 [(*his-72* ^1kb^::HIS-72::GFP); *unc-119(ed3)*] and zuEx181 [(*his-72* ^1kb^::YFP::HIS-72); *unc-119(ed3)*].

### Plasmids.

Genomic sequences containing *his-71* and *his-72* were identified using the Wormbase Web site (http://www.wormbase.org). Plasmids were constructed using the HIS-71::GFP, HIS-72::GFP and YFP::HIS-72 sequences flanked by 1 kb of the gene's own 5′ and 3′ UTRs. A two-step PCR fusion method was used to construct pSO179 (*his-71* ^1kb^::HIS-71::GFP:: *his-71* ^1kb^), pSO159 (*his-72* ^1kb^::HIS-72::GFP:: *his-72* ^1kb^) and pSO186 (*his-72* ^1kb^::YFP::HIS-72:: *his-72* ^1kb^). In the first step, *his-71* and *his-72* sequences were amplified using N2 genomic DNA as template, GFP sequence was amplified from pJH4.52 [36] and YFP sequence was amplified from peYFP (Clontech, Palo Alto, California, United States). In the second step, the three PCR products encoding the *his-71* 5′ UTR::*his-71* ORF, *gfp* and *his-71* 3′ UTR sequences (for pSO179), *his-72* 5′ UTR::*his-72* ORF, *gfp* and *his-72* 3′ UTR sequences (for pSO159) or *his-72* 5′ UTR, YFP and *his-72* ORF::3′ UTR sequences (for pSO186) were fused together by PCR where the middle PCR product contains about 21 to 25 bp overlapping sequences with the first and the third PCR products. Primer sequences for the second step of PCR are ATGATCggtaccTAATTTTTCTTTGGCGATATTTGG and CAAAAGCTGggtaccTAATTCGCCGATTATTTATTCATA for pSO179, ATCGATggtaccAAACGTTATAGTGTGGACACCAATT and AAAGCTGggtaccACGCAACGCGCCGTAAACCTACAC for pSO159, and AAAAGCTGggtaccAAACGTTATAGTGTGGACACCAAT and GGGGGCCCggtaccACGCAACGCGCCGTAAACCTAC for pSO186. The underlined sequences correspond to genomic sequences of *his-71* and *his-72,* introduced restriction sites are in lowercase letters. The fusion PCR product was cloned into pSO160 containing the *unc-119* marker. In pSO159, the linker sequence SRPVAT (TCGAGACCGGTAGCTACT) was used to fuse HIS-72 to GFP. In pSO179 and pSO186, the linker sequence GSRPVAT (GGATCCAGACCGGTAGCTACT) was used to fuse HIS-71 to GFP and YFP to HIS-72, respectively.

### Biolistic bombardment.

Plasmids pSO179, pSO159, and pSO186 were introduced into *unc-119(ed3)* worms to generate HIS-71::GFP, HIS-72::GFP, and YFP::HIS-72, respectively, by performing microparticle bombardment [37]. We obtained transformants with and without germline expressions of *HIS-72* fused to GFP/YFP. The transformants displayed essentially similar somatic expression patterns. The transmission rates of HIS-72::GFP and YFP::HIS-72 are 90% and 70%, respectively. Based on unc− phenotype, HIS-72::GFP is most likely an integrated transgene, and YFP::HIS-72 an extrachromosomal array.

### Microscopy, immunofluorescence, and image analysis.

For the visualization of GFP fluorescence in live animals, worms were transferred to a 10-μl drop of 200 μM levamisole in PBS for 8 min and mounted directly onto coverslips. Live embryos were obtained by cutting open gravid hermaphrodites and mounting them on a coverslip in PBS. GFP fluorescence and light microscopy was performed with a Zeiss Axioplan microscope equipped with epifluorescence, polarizing, and differential interference contrast (DIC) optics.

Worm fixation procedures were performed as described with some modifications [38]. Briefly, gonad dissection was carried out in egg salt buffer (118 mM NaCl, 48 mM KCl, 2 mM CaCl2, 2 mM MgCl2, 5 mM HEPES [pH 7.5]) on a slide. An equal volume of 5% formaldehyde (Electron Microscopy Sciences) in egg salt buffer was added and incubated for 5 min. The sample was freeze-cracked on dry ice, placed into methanol at −20 °C for 5 min, and washed twice in Tris-Tween or PBS. The sample was then stained with DAPI or further processed for antibody staining.

For immunofluorescence, the slide was incubated with primary antibody overnight at 4 °C, secondary antibody for 5 h at room temperature or overnight at 4 °C, stained with DAPI (0.1 μg/ml), and mounted with DABCO (2.3% DABCO in 20 mM Tris [pH 8.0] and 90% glycerol). The following primary antibodies were used: chicken anti-GFP (16901, 1:200 dilution; Chemicon International, Temecula, California, United States), rabbit anti-histone H3K4me2 (07-030, 1:1,000 dilution; Upstate Cell Signaling Solutions, Dundee, United Kingdom) and rabbit anti-PGL-1 (1:10,000 dilution) [25]. The following secondary antibodies were used: Cy3 donkey anti-rabbit antibody (711-165-152, 1:1,000 dilution; Jackson ImmunoResearch Laboratories, West Grove, Pennsylvania, United States) and FITC donkey anti-chicken antibody (703-095-155, 1:200; Jackson ImmunoResearch Laboratories).

To visualize HIS-72::GFP on oocyte chromatin (Figure 6T–6V), we omitted the formaldehyde fixation step. Immunostaining was then carried out as described. The slides were then stored at 4 °C. Image analysis was performed about 3 wk later. We suspect that during storage the nucleoplasmic HIS-72::GFP diffused from the nucleus, making visualization of chromatin-localized HIS-72::GFP possible. In some experiments (Figures 5K–5N, 6A–6D, 6H–6V, and 7A–7T), images were acquired with a scientific grade CCD camera using a wide-field epifluorescence microscope (Olympus) under the control of the DeltaVision system with SoftwoRX (Applied Precision, Mississauga, Ontario, Canada) and processed using SoftwoRX deconvolution software (Applied Precision). Optical sections were collected in 0.20-μm increments. In Figure 6, the presence of an understained chromosome was determined by serial analysis of the optical sections. In Figures 6A–6D, 6H–6S, and 7A–7T, images shown are one section from a z-series collection spaced 0.2 μm apart. In Figures 5K–5N and 6T–6V, images are projections of data stacks generated using Quick Projection from SoftwoRX software. In Figure 5G and 5H, images were collected using a Leica TCS SP Spectral Confocal Microscope. In all figures, images were pseudo-colored.

### High salt extraction of core histones from C. elegans embryos.

Embryos were isolated from gravid HIS-72::GFP hermaphrodites by standard alkaline hypochlorite treatment. The following steps were performed at 4 °C. Nuclei were obtained from embryos using a precooled Dura-Grind stainless steel dounce tissue grinder (Wheaton) with 12.5-μm (0.0005-inch) clearance to homogenize embryos as described [39]. High salt extraction of core histones was performed as described [14]. This method relies on the differential affinities of core histones and nonnucleosomal proteins for DNA at varying salt concentrations. The supernatants were collected at each step. Nuclear pellets were collected by centrifuging at 4,000*g* for 5 min, followed by resuspension in 0.35 M NaCl/1 mM EDTA/10 mM Tris-Cl (pH 8.0) and stirred gently for 15 min. At 0.35 M NaCl, nonnucleosomal proteins are released from DNA. Nuclei were then collected by centrifuging at 2,000*g* for 5 min, resuspended in 0.6 M NaCl/50 mM NaPO_4_ (pH 6.8)/0.2 mM PMSF, and stirred gently for 15 min again. This step lyses the nuclei. Then 20 μl of the sample was used to measure its DNA concentration. Next, hydroxyapatite (130-0520; Bio-Rad, Hercules, California, United States), which binds DNA, was added to the sample to immobilize DNA, based on the ratio of 6.25 mg DNA/mg hydroxyapatite, and stirred gently for another 10 min. The sample was washed twice in the 0.6 M NaCl–containing buffer by centrifuging at 2,000*g* for 5 min. To elute core histones, the nuclei were resuspended in 2.5 M NaCl/50 mM NaPO_4_ (pH 6.8)/0.2 mM PMSF, stirred gently for 15 min, and centrifuged. The sample was washed once in the 2.5 M NaCl–containing buffer. The supernatants from every step were then readjusted to a final concentration of 2.5 M NaCl and frozen at −20 °C. Western blot analysis was performed using rabbit anti-histone H3 core antibody (abcam 1791, 1:5,000 dilution), rabbit anti-GFP antibody (abcam 6556, 1:2,000 dilution), and HRP anti-rabbit (NA9340, 1:10,000 dilution; Amersham Biosciences, Little Chalfont, United Kingdom).

### RT-PCR.

RT-PCR was carried out using SuperScript III reverse transcriptase (Invitrogen, Carlsbad, California, United States). Gene-specific PCR primers were tested for specificity using plasmids encoding the individual H3.3 or H3.3-like genes. The following primers were used: *his-69* (AACGCAATCGTCGTCGGAGCC and TTAGTTGGGCTAGTTGAAT), *his-70* (GTTTTCCCCGTCGACGGACA and CCACGAATTCTTCTAGCCAAC), *his-71* (AGCTCCTCGCAAGCAGCT and GAAACAGCTCAACGTTTAT), *his-72* (CTTCCAGTCGGCTGCCAT and TCGAGAATTGGTGATGGAGC), and *w05B10.1* (AGCTCCCAGAAAAGCTCT and TAAAACAGGGATCTAGCTG).

## Supporting Information

Figure S1 Upon Fertilization, Paternal HIS-72::GFP Appears to Be Lost Temperature-sensitive *fem-1(hc17*ts*) unc-4(e120)* hermaphrodites grown at nonpermissive temperature (23 °C) were crossed to males heterozygous for HIS-72::GFP to determine the fate of paternal HIS-72::GFP. Mating was detected based on the appearance of GFP-containing sperm in the uterus of *fem-1(hc17*ts*) unc-4(e120)* hermaphrodites. However, upon fertilization, paternal HIS-72::GFP appears to be lost. (A, C) GFP fluorescence and (B, D) DIC images of in utero embryos. Scale bars, 10 μm.
(2.2 MB PPT)

Video S1 The X Chromosome Is Deficient in H3.3 during the Pachytene Stage of Oogenesis In adult hermaphrodite pachytene stage nuclei, the X chromosome, which is deficient in H3K4me2, is also deficient in YFP::HIS-72. (A) Anti-GFP (green) and DAPI (red) merged and (B) anti-H3K4me2 (green) and DAPI (red) merged sequential images of pachytene stage nuclei from a dissected and formaldehyde-fixed gonad of an adult hermaphrodite worm. The sequential images were collected at 0.2-μm increments. Scale bars, 5 μM.
(7.6 MB MOV)

Video S2 The X Chromosome Is Deficient in H3.3 during the Pachytene Stage of Spermatogenesis In male pachytene stage nuclei, the X chromosome, which is deficient in H3K4me2, is also deficient in YFP::HIS-72. (A) Anti-GFP (green) and DAPI (red) merged and (B) anti-H3K4me2 (green) and DAPI (red) merged sequential images of pachytene stage nuclei from a dissected and formaldehyde-fixed gonad of a *his-72(tm2066)* male worm. The sequential images were collected at 0.2-μm increments. Scale bars, 5 μm.
(3.1 MB MOV)

### Accession Numbers

The Entrez (http://www.ncbi.nlm.nih.gov/entrez/query.fcgi?DB=pubmed) accession numbers for the proteins shown in Figure 1A are HIS-69 (NP_497811), HIS-70 (NP_497812), HIS-71 (NP_509344), HIS-72 (NP_499608), and w05B10.1/HIS-74 (NP_506164).

## Abbreviations

GFP: green fluorescent protein
RC: replication-coupled
RI: replication-independent
YFP: yellow fluorescent protein
