# Supplementary figures and images for: Histone H3.3 Variant Dynamics in the Germline of Caenorhabditis elegans
Source: PLoS Genet. 2006 Jun 30;2(6):e97. doi: 10.1371/journal.pgen.0020097 (PMC1484599; doi:10.1371/journal.pgen.0020097)

## Slide 1
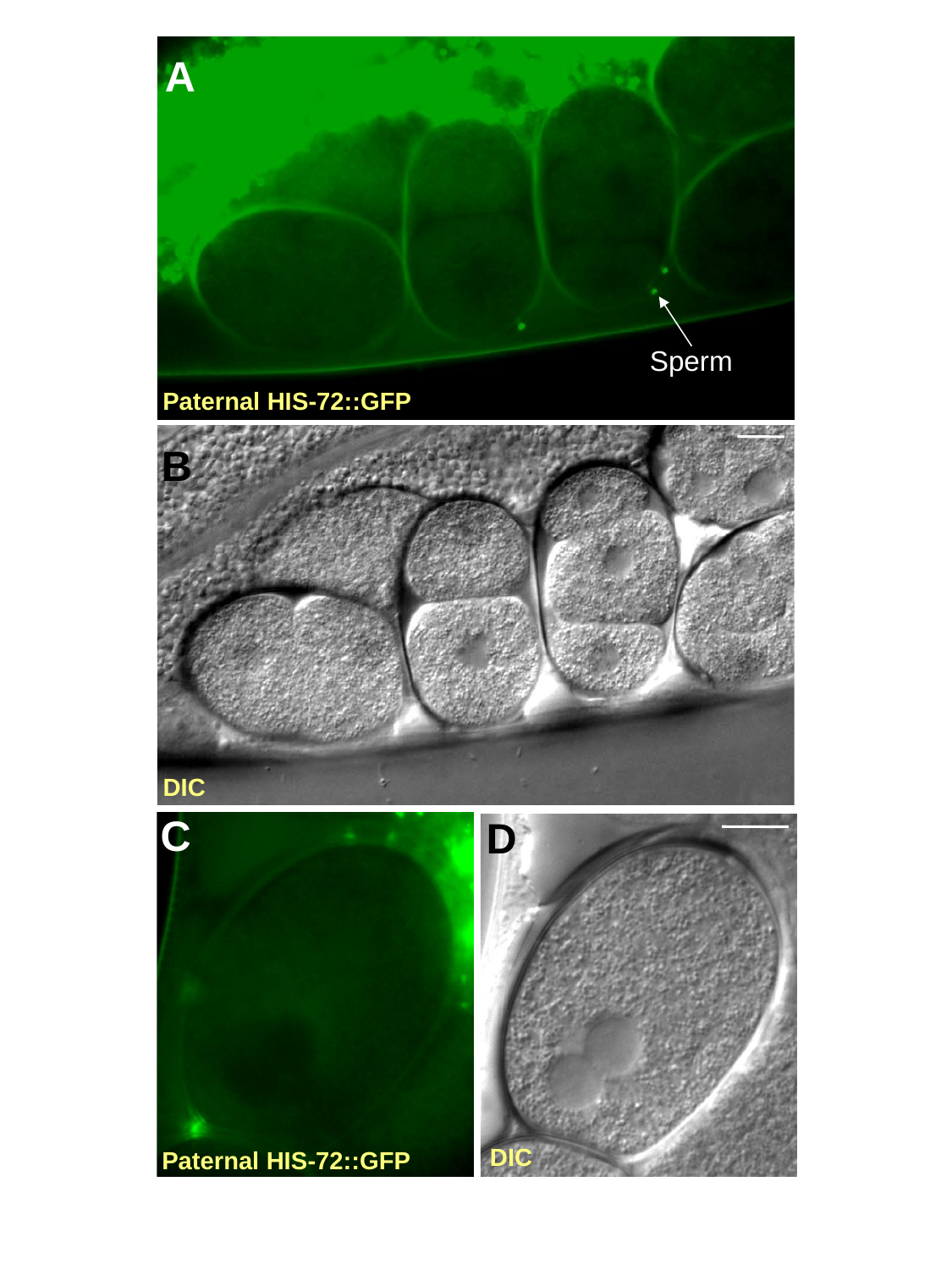

A
Sperm
Paternal HIS-72::GFP
B
DIC
C
D
DIC
Paternal HIS-72::GFP

Supplement: Figure S1 — Temperature-sensitive fem-1(hc17ts) unc-4(e120) hermaphrodites grown at nonpermissive temperature (23 °C) were crossed to males heterozygous for HIS-72::GFP to determine the fate of paternal HIS-72::GFP. Mating was detected based on the appearance of GFP-containing sperm in the uterus of fem-1(hc17ts) unc-4(e120) hermaphrodites. However, upon fertilization, paternal HIS-72::GFP appears to be lost. (A, C) GFP fluorescence and (B, D) DIC images of in utero embryos. Scale bars, 10 μm. (2.2 MB PPT) [file pgen.0020097.sg001.ppt]
